# Outcomes of biological therapy in patients with severe asthma with chronic rhinosinusitis in Saudi Arabia: patients with nasal polyps versus those without nasal polyps

**DOI:** 10.1186/s12890-024-03139-x

**Published:** 2024-07-08

**Authors:** Usama E. Abuelhassan, Medhat Elnamaky, Abdulaziz Alfifi, Sultan K. Kadasah, Mohammed A. Alshehri, Haneen A. Alasiri, Salihah Y. Al-Mani, Ali S. Kadasah, Abdullah Musleh, Fawwaz A. Alshafa, Muhammad S. S. Qureshi, Abdulmohsen Y. Assiri, Abdulrahman I. Falqi, Bader I. Asiri, Haider M. O. Ahmed, Saleem Alshehri, Fasih U. Rahman, Muhammad Amir Qureshi, Omar Abdelwahab, Sherif Mohamed, Ahmed R. I. Ali, Saad M. A. Alqahtani, Abdelrahman M. Abdalla

**Affiliations:** 1 Department of Pulmonary Medicine, Faculty of Medicine, Cairo University, Old Cairo, Cairo Governorate, Cairo, 4240310 Egypt; 2 Department of Internal Medicine, Armed Forces Hospital Southern Region (AFHSR), Khamis Mushayt, Saudi Arabia; 3 Department of Pulmonary Medicine, Faculty of Medicine, Assiut Branch, Al-Azhar University, Assiut, Egypt; 4 Department of Surgery, College of Medicine, University Of Bisha Kingdom of Saudi, Bisha, Saudi Arabia; 5 Otorhinolaryngology Division, Surgery Department, College of Medicine, King Khalid University, Abha, Saudi Arabia; 6 Pharmaceutical Care Administration, Department of Pharmacy, Armed Forces Hospital Southern Region (AFHSR), Khamis Mushayt, Saudi Arabia; 7 Internal Medicine Department, College of Medicine, King Khalid University, Abha, Saudi Arabia; 8 College of Medicine, Al Faisal University, Riyadh, 11533 Saudi Arabia; 9 Department of Chest Diseases and Tuberculosis, Faculty of Medicine, Assiut University, 71516 Assiut, Egypt; 10 Department of Internal Medicine, Sultan Bin Abdulaziz Humanitarian City, Riyadh, 13571 Saudi Arabia; 11 Department of Anesthesia, Intensive Care and Pain Management, Faculty of Medicine, Al Azhar University, Cairo, Egypt; 12 Department of Adult Intensive Care, Armed Forces Hospital Southern Region (AFHSR), Khamis Mushayt, Saudi Arabia; 13 Department of Pulmonary Medicine, Faculty of Medicine, Beni Seuf University, Beni Seuf, Egypt

**Keywords:** Outcomes, Rhinosinusitis, Nasal polyps, SNOTT-22, Clinical, Severe asthma, Saudi Arabia, Comparison

## Abstract

**Background:**

This study’s purposes were to evaluate the impact of biological therapies on outcomes in patients with severe asthma (SA) and chronic rhinosinusitis (CRS) and to compare these effects among those with NP (CRSwNP) versus those without NP (CRSsNP) in the “real-world” setting in Saudi Arabian patients.

**Methods:**

From March to September 2022, a retrospective observational cohort study was undertaken at the severe asthma clinics of the Armed Forces Hospital—Southern Region (AFHSR) and King Khalid University Hospital, Abha, Saudi Arabia, to delineate the effects of dupilumab therapy. Outcomes were assessed, including clinical outcomes, FEV1, and laboratory findings before and one year after dupilumab. Post-therapy effects were compared between CRSwNP and CRSsNP.

**Results:**

Fifty subjects were enrolled, with a mean age of 46.56. There were 27 (54%) females and 23(46%) males. Significant improvements in clinical parameters (frequency of asthma exacerbations and hospitalizations, the use of OCs, anosmia, SNOTT-22, and the ACT), FEV1, and laboratory ones (serum IgE and eosinophilic count) were observed 6 and 12 months after using dupilumab (*p* < 0.001), respectively. However, after 12 months of dupilumab therapy, there were no significant differences between those with and without NP with regards to clinical (anosmia, ACT, and OCs use), laboratory (eosinophilic count, serum IgE level) parameters, and FEV1%.

**Conclusions:**

Patients with CRS experienced significant improvements in clinical, FEV1, and laboratory outcomes after dupilumab therapy. However, these improvements were not maintained when comparing CRSwNP with CRSsNP. There were no significant differences between those with and without NP regarding ACT and OCs use or laboratory (eosinophilic count, serum IgE level) parameters. Further prospective multicenter studies are warranted.

## Background

The definition of severe asthma (SA) is “asthma that requires therapy with high-dose inhaled corticosteroids (ICS) plus a second controller (e.g., long-acting beta-2 agonist (LABA), long-acting muscarinic antagonist (LAMA), leukotriene modifier and/o oral corticosteroids (OCS) to prevent it from becoming “uncontrolled” or that remains uncontrolled despite such therapy” [1, 2]. Severe asthma (SA) affects 3–10% of asthma patients and is associated with increased mortality, hospitalization, decreased quality of life, and higher healthcare costs [1].

Chronic rhinosinusitis with nasal polyposis (CRSwNP) coexists in over 30% of persons with severe asthma, with or without aspirin-exacerbated respiratory disease (AERD) [3]. CRSwNP has a high rate of recurrence after sinonasal surgery, can be refractory to topical nasal therapies, and can be effectively treated by biologics, with dupilumab, omalizumab, and mepolizumab having a regulatory indication separate from asthma [4, 5].

The combination of severe asthma with chronic rhinosinusitis (CRS), particularly CRS with nasal polyposis (CRSwNP), presents a unique phenotype, and the relationship between asthma and CRSwNP is not just a simple association. Core pathophysiological mechanisms are shared, with T2 inflammation being the cornerstone of these disorders. Thus, taking into consideration that this T2 inflammation strongly impacts the symptoms and burdens of both diseases, one can expect that patients who have severe asthma will often experience severe CRSwNP symptoms, too, and vice versa [6].

On the other hand, chronic rhinosinusitis is divided into two major macroscopic phenotypes according to the presence (CRSwNP) or absence (CRSsNP) of nasal polyps [7]. One may expect a better response to biologic treatment in patients with SA and CRSwNP. This is evident in reducing asthma exacerbations, using maintenance steroids, and improving lung function, control, and quality of life [6, 8].

However, this scenario is only sometimes the case. Some reports have seen no significant differences in the effects of biological therapies between patients with CRS with nasal polyps (CRSwNP) and those without (CRSsNP) [9, 10]. Even more, some data went beyond and claimed that the phenotypic classification of CRS into those with and without nasal polyps is inadequate [11, 12].

Many worldwide studies have addressed the impact of biological therapies in patients with SA combined with CRS [8, 10]. However, no studies addressed that issue in Saudi Arabian patients. Moreover, no Saudi studies have compared the effects of biological therapy in patients with SA and NP (CRSwNP) vs. those without NP (CRSsNP).

Therefore, the current study’s purposes were to evaluate the impact of biological therapies on outcomes in patients with SA and CRS and to compare these effects among those with SA and NP (CRSwNP) versus those without NP (CRSsNP) in the “real-world” setting in Saudi Arabian patients.

## Materials and methods

### Study design and population

The current research is a retrospective observational cohort study that was undertaken at the severe asthma clinics of the Armed Forces Hospital—Southern Region (AFHSR) and King Khalid University Hospital, Abha, Saudi Arabia, from March to September 2022. This study aimed to delineate the effects of biological therapies in adults with severe eosinophilic asthma and concomitant rhinosinusitis and compare these effects between patients with SA and CRSwNP and SA with CRSsNP.

### Inclusion and exclusion criteria

Participants were adults (≥ 18 years) diagnosed with SA as per the diagnostic criteria of the Global Initiative for Asthma; GINA 2023 guidelines [1] and concomitant rhinosinusitis, meeting criteria from Orlandi et al. [2]. Exclusion criteria were chest X-ray abnormalities suggestive of interstitial lung disease (ILD), Type 2 low asthma, patients with allergic bronchopulmonary aspergillosis (ABPA), patients with eosinophilic granulomatosis with polyangiitis (EGPA) or having positive anti-nuclear cytoplasmic antibodies (ANCA), patients with hemoglobin < 10 g/dl, those with significant cardiac or autoimmune conditions, fixed or irreversible airway obstruction, paradoxical vocal fold motion, and those with documented history or high resolution computed tomography (HRCT) findings of bronchiectasis or ILD.

### Assessments

Chronic rhinosinusitis (CRS) was assessed using the criteria from Orlandi et al. [2] and divided into two phenotypes according to the presence (CRSwNP) or absence (CRSsNP) of macroscopic nasal polyps, respectively [7].

Asthma Exacerbations: we defined exacerbations as episodes with worsened respiratory symptoms and decreased lung function requiring treatment alteration, in alignment with the American Thoracic Society/European Respiratory Society (ATS/ERS) statements [13].

Clinical Assessment: Routine clinic evaluations included biannual serum eosinophils, IgE measurements, and pulmonary function tests (PFTs). ACT scores were recorded semiannually and retrieved from the patient’s medical records. Obesity was defined as body mass index, BMI ≥ 30 kg/m2 [14].

ACT: ACT scores, ranging from 5 to 25, assessed asthma control levels, with higher scores indicating better management [15].

Oral corticosteroids (OCs) use: OCs use was referred to any corticosteroid prescription filled during the study’s maintenance or exacerbation management time frame, averaged from pharmacy dispensation records to quantify systemic exposure.

Sense of smell: the patient’s sense of smell was assessed subjectively.

Sino-nasal outcome test-22 (SNOTT-22): The SNOT-22 is a validated, self-administered questionnaire to assess CRS patients [16]. It comprises 22 items, rated from 0 (‘no problem at all’) to 5 (‘worst possible symptom’). Thus, possible overall scores range from 0 to 110, with higher scores indicating worse symptoms. Scores are stratified into mild (sores 8–20), moderate (> 20–50), and severe (> 50) [16].

Biological therapy indication: biological therapy followed the ERS/ATS 2020 recommendations [17], with the anti-IL-5 benralizumab initiated at eosinophil counts ⩾150 µL − 1 and omalizumab considered at counts ⩾260 µL − 1. Dupilumab served as an adjunct for those inadequately controlled on conventional regimens.

Dose of dupilumab: Dupilumab was administered as a subcutaneous loading dose of 600 mg, followed by 300 mg every two weeks [1].

### Outcome measures and data collection

Data encompassing demographics, clinical evaluations, and treatment histories were systematically extracted from electronic health records for analysis.

### Ethical considerations

The Armed Forces Hospital Southern Region (AFHSR) Institutional Review Board (IRB) approved the study, with approval number AFHSRMREC/2022/PULMONOLOGY-INTERNAL MEDICINE/681.

### Statistical analysis

Descriptive statistics were presented as mean ± SD for normally distributed variables and using median (IQR) for non-normally distributed variables, while frequencies and percentages were used with categorical variables. The three biological treatment groups were compared using One-way ANOVA or the Kruskal-Wallis test for numerical variables. In contrast, the Chi-square test was utilized for categorical variables. Treatment response before biological therapy, six months, and 12 months after biologic therapy was compared using repeated measures ANOVA for numerical variables, or Cochrane Q test for categorical variables, while the comparison between pre-treatment and 12 months after was done using paired-samples t-test, Wilcoxon signed rank test or Mcnemar test. *P*-value < 0.05 is statistically significant, and IBM SPSS for Windows version 29 was used for the statistical analysis.

### Clinical trial number

This study was not registered in any clinical trial registry and does not have a clinical trial number.

## Results

### Demographic and clinical characteristics before biological therapy

Fifty-six patients were enrolled in the current study. Fifty patients received dupilumab, three received omalizumab, and three received benralizumab. The small numbers of those who received omalizumab and benralizumab were excluded to avoid the affection of statistical analysis. So, 50 patients who received dupilumab were enrolled, with a mean age of 46.56 ± 13.93 years, and they were 27 (54%) females and 23(46%) males. The mean body mass index (BMI) was 30.52 ± 4.27 kg/m2, with obesity found in 29 (58%) patients. Twenty-eight (56%) patients had nasal polyps, while 22 (44%) had no NP. The following most common comorbidities were gastro-oesophageal reflux disease, GERD (19/50, 38%), anxiety (16/50, 32%), and obstructive sleep apnea; OSA (6/50, 12%), respectively. Before biological therapy, all the study subjects received the standard treatments for severe asthma. All patients received high-dose ICs, LABA, and LAMA. Remarkably, all patients received OCs. Among those patients with NP (*n* = 28), 25 underwent one or more surgeries, whereas 3 refused surgical management. (Table 1)

**Table 1:** Baseline demographics and comorbidities of the enrolled patients (*N* = 50)

		*N* (%)
Age	Mean ± SD	46.56 ± 13.93
	Min - Max	18–80
Sex	Male	23 (46%)
	Female	27 (54%)
BMI	Mean ± SD	30.52 ± 4.27
	Min - Max	19–42
Obesity	No	21 (42%)
	Yes	29 (58%)
Smoking	Active Smoker	3 (6%)
	Non/Former Smoker	47 (94%)
Pre IgE	Mean ± SD	405.43 ± 291.09
	Min - Max	21–1150
Pre Eosinphiles	Mean ± SD	657.79 ± 363.24
	Min - Max	100–1490
Comorbidities		
GERD		19 (38.0%)
Anxiety		16 (32%)
ACO		3 (6%)
Chronic rhinosinusitis		50 (100%)
OSA		6 (12%)
Nasal polyps		28 (56.0%)
FEV1%	Mean ± SD	53.58 ± 9.05
	Min - Max	33–71
Asthma medications before biological therapy		N (%)
High ICS		50 (100%)
LABA		50 (100%)
LAMA		50 (100%)
OCS		50 (100%)
Exacerbations/year	Mean ± SD	2.28 ± 0.95
	Min - Max	1–5
BA duration in years	Mean ± SD	9.26 ± 5.28
	Min - Max	1–25

BMI: Body mass index, GERD: gastroesophageal reflux disease, ACO: Asthma-COPD overlap, OSA: obstructive sleep apnea, OCs: oral corticosteroid, ICS: inhaled corticosteroids, LABA: long-acting beta-2 agonist, LAMA: long-acting muscarinic antagonist

### Treatment response (before and after dupilumab)

The following parameters were compared before and 6 and 12 months after using dupilumab: clinical parameters (frequency of asthma exacerbations and hospitalizations, the use of OCs, anosmia, SNOTT-22, and the ACT), FEV1, and laboratory parameters (serum IgE and eosinophilic count).

Characteristically, there were significant improvements in all these parameters. There was a substantial decrease in the frequency of exacerbations and hospitalization, anosmia, Ocs use, and SNOTT-22 scores after 6 &12 months of dupilumab therapy compared to pre-biological therapies, respectively (*p* < 0.001, each). The mean ACT scores increased significantly to 18.13 ± 1.55 and 19 ± 1.85 at 6 &12 months post-biological treatment, respectively, compared to 13.63 ± 2.62 pre-biological therapy (*p* < 0.001). There was a significant increase in FEV1% from 51.45 ± 10.10 to 65.2 ± 8.8 and 67.29 ± 7.38 before, 6, and 12 months after dupilumab therapy (*p* = 0.004), respectively. There were significant decreases in serum IgE and eosinophilic counts from 505.48 ± 317.70 IU/ml and 514.44 ± 270.38 µL − 1 before therapy to 147.54 ± 180.76 IU/ml, 101.19 ± 95.3 IU/ml, and 492.41 ± 376.19 µL − 1 and 282.7 ± 192.71 µL − 1, after 6 and 12 months of dupilumab therapy, (*p* < 0.001, each), respectively. (Table 2 details these results)

**Table 2:** Treatment outcomes before, 6, and 12 months after dupilumab therapy (*N* = 50)

	Before dupilumab	Six months after dupilumab	12 months after dupilumab	*P*-value
ACT	13.63 ± 2.62^a^	18.13 ± 1.55^b^	19 ± 1.85^b^	< 0.001
Frequency of exacerbation	2 (1)		0 (0)	< 0.001
Frequency of hospitalization	1 (0)		0 (0)	< 0.001
OCs use, N(%)	50 (100%) ^a^	6 (12%) ^b^	2 (4%) ^b^	< 0.001
Anosmia	2 (9.1%)		0 (0.0%)	< 0.001
SNOT22 score	38.75 ± 18.37		15.13 ± 9.38	< 0.001
FEV1%	51.45 ± 10.10	65.2 ± 8.8	67.29 ± 7.38	0.004
Eosinophils	514.44 ± 270.38	492.41 ± 376.19	282.7 ± 192.71	< 0.001
IgE	505.48 ± 317.70	147.54 ± 180.76	101.19 ± 95.3	< 0.001

Different superscript letters indicate statistically significant differences upon comparison using post-hoc analysis

Dupilumab was well tolerated, and the most common side effects in our cohort include injection-site reactions (24/50, 48%) and peripheral blood eosinophilia(13/50, 26%). No severe side effects have been reported that require cessation of treatment.

### Clinical & laboratory differences between patients with and without NP

Before starting dupilumab therapy, there were significant differences between patients with NP and those without NP. Compared to patients without NP, those with NP had higher mean eosinophilic count (770.43 ± 390.76 vs. 514.44 ± 270.38, *p* = 0.009), mean SNOTT scores (69.39 ± 16.77 vs. 38.75 ± 18.37, *p* ≤ 0.001), and higher percentages of anosmia (39.3% vs. 9.1%, *p* = 0.016). In contrast, they had lower serum IgE levels (326.82 ± 246.39 vs. 505.48 ± 317.70, *p* = 0.024), respectively. On the other hand, no significant differences were encountered between the two groups regarding gender, age, BMI, percentages of associated comorbidities, asthma duration, number of exacerbations per year, and FEV1. (Table 3)

**Table 3:** Clinical characteristics and laboratory findings according to having nasal polyps or not

		No nasal polyps (*N* = 22)	Nasal polyp (*N* = 28)	*P*-value
Age (years)	Mean ± SD	44.86 ± 12.78	47.89 ± 14.86	0.451^+^
BMI	Mean ± SD	30.67 ± 3.20	30.41 ± 5.01	0.835^+^
Sex	Male	10 (45.5%)	13 (46.4%)	0.945^+++^
	Female	12 (54.5%)	15 (53.6%)	
Comorbidities	Yes	14 (63.6%)	20 (71.4%)	0.558^+^
Exacerbations/year (pre)	Mean ± SD	2.14 ± 0.71	2.39 ± 1.10	0.348^+^
BA duration (years)	Mean ± SD	8.68 ± 5.58	9.71 ± 5.08	0.498^+^
Pre Eosinophils	Median (IQR)	450.0 (451.25)	689.0 (640.5)	0.020^++^
Pre IgE	Mean ± SD	505.48 ± 317.70	326.82 ± 246.39	0.024^++^
Pre FEV1%	Median (IQR)	55.0 (16.25)	55.0 (13.25)	0.357^++^

^+^ Independent t-test test was used, ^++^ Mann-Whitney test was used ^+++^ Chi-square test was used, * P-

### Effects of dupilumab on patients with and without NP

At six months after dupilumab therapy, there were no significant differences between those with and without NP with regards to clinical (anosmia, ACT, OCs use, and SNOTT22 score), laboratory parameters (eosinophilic count, serum IgE level), and FEV1%.

After 12 months of dupilumab therapy, there were no significant differences between those with and without NP with regards to clinical (anosmia, ACT, and OCs use), laboratory (eosinophilic count, serum IgE level) parameters, and FEV1%. Only there was a significant difference with regards to the SNOTT-22 score; those with NP had higher scores (26.50 ± 12.53) compared to those without NP (15.13 ± 9.38), *p* ≤ 0.001, respectively. (Table 4)

**Table 4:** Comparisons between patients with and without nasal polyps before, 6, and 12 months after dupilumab therapy

	Before treatment			At 6 m post-treatment			At 12 m post-treatment		
	**No nasal polyps (** *N* ** = 22)**	**Nasal polyp (** *N* ** = 28)**	***P-value***	**No nasal polyps (** *N* ** = 22)**	**Nasal polyp (** *N* ** = 28)**	***P-value***	**No nasal polyps (** *N* ** = 22)**	**Nasal polyp (** *N* ** = 28)**	***P-value***
	Median (IQR)	Median (IQR)					Median (IQR)	Median (IQR)	
Frequency of exacerbation	2 (1)	2 (1)	0.607				0 (0)	0 (0)	0.429
Frequency of hospitalization	1 (1.3)	1 (1)	0.567				0 (0)	0 (0)	0.205
	Mean ± SD	Mean ± SD		Mean ± SD	Mean ± SD		Mean ± SD	Mean ± SD	
FEV1%	51.45 ± 10.10	55.25 ± 7.91	0.134	65.2 ± 8.8	65.04 ± 9	0.948	67.29 ± 7.38	66.85 ± 6.95	0.83
ACT	13.40 ± 2.63	12.94 ± 2.12	0.499	20.36 ± 3.09	18.6 ± 3.07	0.051	20.19 ± 2.99	18.77 ± 2.77	0.088
Eosinophils	514.44 ± 270.38	770.43 ± 390.76	0.009	492.41 ± 376.19	378.21 ± 357.56	0.279	282.7 ± 192.71	306.03 ± 206.55	0.685
IgE	505.48 ± 317.70	326.82 ± 246.39	0.024	147.54 ± 180.76	119.67 ± 121.24	0.518	101.19 ± 95.3	92.12 ± 96.1	0.741
SNOT22 score	38.75 ± 18.37	69.39 ± 16.77	< 0.001				15.13 ± 9.38	26.50 ± 12.53	< 0.001
	N (%)	N (%)		N (%)	N (%)		N (%)	N (%)	
OCS use	22 (100%)	28 (100%)		2 (9.1%)	4 (14.3%)	0.683	0 (0.0%)	2 (7.1%)	0.497
Anosmia	2 (9.1%)	11 (39.3%)	0.016				0 (0.0%)	3 (10.7%)	0.246

## Discussion

To the best of our knowledge, this is the first real-life study to address the effectiveness of biological therapy (dupilumab) among Saudi Arabian patients with SA and CRS and compare this effectiveness in CRS patients with NP (CRSwNP) vs. those without NP (CRSsNP).

Asthma is a heterogeneous disease that affects more than 2 million in Saudi Arabia, and the majority of them have uncontrolled asthma, with an affection for their quality of life. A recent meta-analysis [18] has shown that the pooled weighted prevalence rates of asthma and rhinitis in Saudi Arabia were 14.3% and 21.4%, respectively, with an increase in asthma prevalence from 1990 to 2000 and a stabilized or not-so-significant decline from 2010 to 2016 was observed [18].

The current study was a real-world study that followed patients with severe asthma who received biological therapies for 12 months. This was a good follow-up duration, giving us robust data about the response to dupilumab therapy regarding clinical improvements. Interestingly, previous studies had shorter follow-up durations [19].

The use of biological therapy was a necessity for our cohorts. All the study subjects received standard asthma medications, yet their asthma was uncontrolled. Moreover, 25/28 (89.2%) of CRSwNP underwent surgery. Thus, the enrolled subjects were candidates for biological therapies. On the other hand, the results of the current study reflect the role and magnitude of T2 inflammation in our cohorts with SA and CRS.

Thus, our results significantly improved our cohorts’ clinical, FEV1, and laboratory outcomes. Overall, we observed significant decreases in the frequency of exacerbations and hospitalization, anosmia, Ocs use, and SNOTT-22 scores, a significant increase in the ACT scores and FEV1%, and significant decreases in serum IgE and eosinophilic counts after 6 &12 months of dupilumab therapy compared to pre-biological treatment, respectively. Our results emphasize the importance of eosinophilic inflammation in patients with SA and CRS and agree with those published previously and show that biologics that target T2 inflammatory pathways are highly effective in achieving asthma control and reducing the risk of exacerbations in those patients with T2 inflammation whose asthma is uncontrolled with moderate to high doses of ICS and additional controller therapies [1, 10, 17, 18]. Also, our results agree with those meta-analyses and real-wide studies that reported such improvements with individual biologics [19–21]. Moreover, dupilumab is the only asthma biologic with a specific regulatory indication for OCs-dependent asthma without a biomarker requirement. This creates practical advantages as blood eosinophilia can be masked in chronic maintenance OCs [19].

Chronic rhinosinusitis with nasal polyposis (CRSwNP) coexists in over 30% of persons with severe asthma, with or without aspirin-exacerbated respiratory disease (AERD) [3]. Chronic rhinosinusitis is divided into two major macroscopic phenotypes according to the presence (CRSwNP) or absence (CRSsNP) of noncancerous growths in the lining of the nose and surrounding sinuses (i.e., nasal polyps) [7]. Most cases in Europe and North America are labeled with a chronic type 2 inflammatory response with tissue eosinophilia and significantly elevated levels of tissue expression of IL-5 and CLC [22]. CRSwNP in Asia was historically described as a predominantly neutrophilic disease; however, in the last 20 years, a shift toward an increased proportion of patients with tissue eosinophilia has been documented in several Asian countries [23].

Pathophysiologically, eosinophils accumulate and display evidence of prolonged survival in sino-nasal mucosae of patients with CRSwNP and release into the tissue several inflammatory mediators that are thought to be, at least partially, responsible for many of the pathological features and clinical consequences of the chronic inflammation [22, 24]. Moreover, eosinophils from nasal polyps are activated and can promote innate and adaptive immune responses, fibrin formation, and tissue remodeling, directly contributing to CRSwNP pathogenesis [22, 25].

Our results showed that before starting dupilumab therapy, compared to patients without NP, those with NP had higher mean eosinophilic count, mean SNOTT scores, and higher percentages of anosmia. In contrast, they had lower serum IgE levels. These findings reflect the impact of T2 inflammation in patients with SA combined with CRSwNP regarding clinical symptoms and laboratory findings. Previous reports have demonstrated that dupilumab was more effective in patients with T2 asthma characterized by elevated levels of eosinophils or FeNO, usually *>* 25 ppb [26, 27].

On the other hand, a comparison between patients with CRSwNP and those with CRSsNP revealed exciting results.

After 12 months of dupilumab therapy, no significant differences existed between those with and without NP regarding clinical (anosmia, ACT, and OCs use), laboratory (eosinophilic count, serum IgE level) parameters, and FEV1%. Only the SNOTT22 score was significantly different, with those with NP having higher scores than those without NP. These results could be explained in many ways. First, the relatively low number of enrolled subjects among both CRSwNP and CRSsNP groups in the current study may not reflect precise outcome results between the two groups. Second, as a biomarker for eosinophilic diseases, sputum eosinophils are more accurate than blood eosinophils. We assessed only blood eosinophils, which could not be exclusively representative of the magnitude of the eosinophilic inflammation in our cohorts. Third, data has increasingly emerged that the phenotypic classification of CRS into those with and without nasal polyps needs to be improved [11, 12]. Phenotypes of CRS do not necessarily conform to presumed histopathologic and endotypic characteristics. Many patients with CRSsNP have eosinophilic infiltration and likely have type 2-driven immunopathology [12, 28]. Conversely, many patients with CRSwNP may not have the presumed type 2 inflammation and, therefore, may respond poorly to biologic therapy [29].

Recent trials of dupilumab and omalizumab report a polyp size decrease of about 2 points on an 8-point total nasal polyp score in CRSwNP patients [4, 5]. Fourth, the finding of significant differences only in SNOTT-22 scores between the two groups could be explained by the fact that dupilumab might have higher effects on nasal symptoms and SNOTT-22 scores in patients with SA and CRSwNP. This agrees with previous RCTs that addressed the role of dupilumab as an add-on therapy to nasal corticosteroids in CRwNP [4, 8]. Moreover, the current study’s results agree with those of Förster-Ruhrmann and colleagues [10]. In their study, the authors addressed the pulmonary and nasal outcomes in patients with SA and nasal polyposis. In a retrospective study, they enrolled 115 adult patients with SA and CRSwNP who received 1 of the four biologics (mepolizumab, benralizumab, dupilumab, omalizumab). Outcomes were evaluated by Asthma Control Test (ACT), FEV1%, GINA-severity grade, rhinological questionnaires (CRS visual analog scale (VAS) scores, and sinonasal QoL Rhinosinusitis Outcome Measure-31 (RSOM-31) before and after 4–6 months of therapy.

Interestingly, the authors found that the most significant differences in the pre/post scores were encountered in the patients who received dupilumab, with the most notable improvement for all nasal symptoms and scores. However, there were no significant changes in the scores for patients in the benralizumab and mepolizumab groups [10].

From a practical point of view, our study highlights the need for further direct therapy based on the endoscopic visualization of the presence or absence of polyps and the biological characteristics of CRS. Trials should utilize criteria beyond the CRSwNP phenotype, using biomarkers and other clinical data. These could include histopathology data, tissue eosinophil count [12, 30], or other biomarkers under investigation [31,32]. Moreover, more studies are needed to address the magnitude of eosinophilic inflammation in patients with CRSsNP [12]. More specific inclusion criteria should be more relaxed in prior trials of biologics and need to reflect real-world scenarios. Biologic therapy could be an option for all eosinophilic CRS, mainly if used for a labeled condition of uncontrolled asthma.

Our study has many strengths. It is the first real-life study that addressed the effectiveness of biological therapy (dupilumab) among Saudi Arabian patients with SA and CRS and compared this effectiveness in patients with (CRSwNP) vs. (CRSsNP). The one-year follow-up period was more extended than that of most similar studies. However, our study had several limitations. It is a retrospective study, and it is affected by the limitations of a retrospective study—data on tissue eosinophilia and structured histopathological reports needed to be included. Instead, this study highlights the need to conduct further prospective studies in Saudi Arabia on the use of biologic therapy on CRS patients in a real-world setting, including patients who still need to meet past enrollment criteria for clinical trials.

## Conclusion

This is the first real-life study from two large Saudi Arabian tertiary centers for the effects of dupilumab therapy in patients with severe asthma and chronic rhinosinusitis that compared CRwNP versus CRsNP. There were significant improvements in patients with CRS after dupilumab therapy regarding clinical, FEV1, and laboratory outcomes. However, these improvements were not maintained when comparing CRSwNP with CRSsNP. There were no significant differences between those with and without NP regarding ACT and OCs use or laboratory (eosinophilic count, serum IgE level) parameters. Further prospective multicenter studies are warranted.

## Data Availability

The datasets used or analysed during the current study are available from the corresponding author on reasonable request.

## Declarations

### Clinical trial number

This study was not registered in any clinical trial registry and does not have a clinical trial number.
